# The effects of combined spinal-epidural analgesia and epidural anesthesia on maternal intrapartum temperature: a randomized controlled trial

**DOI:** 10.1186/s12871-022-01898-w

**Published:** 2022-11-15

**Authors:** Zhiping Yao, Jingxin Zhou, Shuying Li, Wenqin Zhou

**Affiliations:** Department of Anesthesiology, West China Second Hospital of Sichuan University, Key laboratory of Birth Deficits and related Diseases of Women and children, Sichuan University, Ministry of education, No.20, Section 3, Renmin Nanlu, Chengdu, China

**Keywords:** Combined spinal-epidural analgesia, Epidural anesthesia, Maternal intrapartum temperature, Fever

## Abstract

**Background:**

Labor epidural analgesia has been suggested to be associated with intrapartum fever. We designed this study to investigate the effects of epidural analgesia and combined spinal-epidural analgesia on maternal intrapartum temperature.

**Methods:**

Four hundred healthy nullipara patients were randomly assigned to receive either epidural analgesia (EA group) or combined spinal-epidural analgesia (CSEA group). Maternal temperature was measured hourly after analgesia administration. The primary outcome was the incidence of maternal fever, and the secondary outcomes were the duration of analgesia, analgesia to full cervical dilation and analgesia to delivery. Neonatal outcomes and other basic labor events were also recorded.

**Results:**

Maternal temperature gradually increased with time in both analgesia groups during labor. However, the CSEA group had a lower incidence of maternal fever, and a lower mean maternal temperature at 5 h, 6 h, and 9 h after analgesia. In addtion, the CSEA group also had a shorter time of analgesia duration, analgesia to full cervical dilation, analgesia to delivery, and less dose of epidural local anesthetic than the EA group.

**Conclusion:**

Our findings suggest that combined spinal-epidural analgesia is associated with a lower risk of intrapartum fever than epidural analgesia.

**Trial registration:**

ChiCTR1900026606. Registered on 16/10/2019.

## Introduction

Epidural analgesia (EA) has been widely used during labor for its effective and safe analgesic effects. However, both randomized trials and observational studies have indicated that labor epidural analgesia is associated with the development of maternal fever or temperature elevation [1–4]. The risk of epidural-related fever has been reported to range from 15 to 42% in nulliparas [4–8]. Maternal intrapartum fever is considered to be a risk factor for both the mother and the neonate, including an increased amount of unnecessary maternal antibiotic use, and number of neonatal evaluations for sepsis and other adverse neonatal outcomes [9, 10].

Several studies have reported that maternal intrapartum fever is associated with epidural analgesia, prolonged labor, prolonged membrane rupture, increased vaginal examinations, high body mass index (BMI), high gestational age, and nulliparity [11–15]. The duration of labor is a very significant predictor of maternal intrapartum fever and can be partially controllable. Although epidural-related fever is a hot topic in obstetrics, there is no effective method to prevent. Combined spinal-epidural analgesia (CSEA) is used as an alternative form of epidural analgesia, with a faster onset of pain relief and higher maternal satisfaction. One study reported that CSEA was associated with more rapid cervical dilation and shorter time to full cervical dilation than epidural analgesia in healthy nulliparity [16]. Therefore, we hypothesized that CSEA is more effective in reducing the maternal temperature that epidural analgesia in labor. The aim of this study was to explore the possible effects of CSEA and EA on maternal intrapartum temperature.

## Methods

This study was approved by the China Ethics Committee of Registering Clinical Trials (ChiCTR1900026606) and was registered in the Chinese Clinical Trial Registry. After registering the study, and obtaining written informed consent from all patients, we started this prospective, randomized, controlled, non blinded study. The trial was conducted between October 2019 and February 2020.

The inclusion criteria were healthy nulliparity, age ≥ 20 years, singleton cephalic presentation, term pregnancy (≥37 weeks), cervical dilatation of 1–5 cm, and request for labor neuraxial analgesia. Parturients who had a baseline temperature ≥ 37.5 °C, contraindications to labor neuraxial analgesia, nonreassuring fetal heart rate tracings, or high-risk pregnancies (preeclampsia, placenta previa, known fetal anomalies, severe system comorbidities) were excluded. In addition, parturients who required cesarean delivery within 2 h after analgesia or experienced failed labor neuraxial analgesia were also excluded from this study.

Parturients were randomly allocated to two equal groups using computer-generated random numbers: the CSEA group and the EA group. The chief anesthesia resident with more than 3 years of obstetric anesthesia experience administered labor analgesia. The parturients were coloaded with 500 ml lactated Ringer’s solution and placed in the left lateral position. A convenient lumbar epidural space was located and identified by a 17-gauge Tuohy needle using a loss of resistance to air technique. In the CSEA group, a 26-gauge pencil-point spinal needle perforated the dura using the needle-through-needle technique, and a subarachnoid injection with bupivacaine 2.5 mg with fentanyl 15 μg was performed. After intrathecal injection, a multiorifice epidural catheter was inserted 3–5 cm into the epidural space and secured. If a bilateral T-10 sensory level was not attained or VAS score > 3 within 20 min, CSEA was defined as failed, and the patient was excluded from the study. For the successful CSEA, when the sensory level block down to the T10, a test dose of 3 mL 1.5% lidocaine with epinephrine 1:200,000 was administrated into the epidural space to ensure that an intravascular or subarachnoid puncture was not observed. A bolus of 8–10 ml 0.1% ropivacaine and 0.5 μg/ml sufentanil was administered, and a continuous infusion at a rate of 8 ml/h was established to maintain the sensory block at T10 or higher. If a bilateral T10 sensory level was not attained within 20 min after the epidural injection, an additional 5–10 ml was administered. If a bilateral T10 sensory level was still not attained after a total of 20 ml was administered, CSEA was also defined as failed.

In the EA group, a multi-orifice epidural catheter was directly inserted 3–5 cm into the epidural space, and a test dose of 3 mL 1.5% lidocaine with epinephrine 1:200,000 was administrated. After ensuring that the epidural catheter was not located in the intravascular or subarachnoid space, a bolus of 8–10 ml 0.1% ropivacaine and 0.5 μg/ml sufentanil was administered, and a continuous infusion was maintained at a rate of 8 ml/h to maintain the sensory block at T10 or higher. If a bilateral T10 sensory level was not attained within 20 min after the epidural injection, an additional 5–10 ml was administered. If a bilateral T10 sensory level was still not attained after administering a maximum of 20 ml, epidural analgesia was defined as failed.

After labor neuraxial analgesia, routine maternal and fetal monitoring was continued. The delivery room temperature was maintained between 20 °C and 22 °C. Maternal temperature was measured before analgesia and hourly after analgesia with an electronic thermometer inserted into the ear canal near the tympanum until delivery. The anesthesia nurse was followed up every 1–2 hours and collected the data. Intrapartum fever was defined as an tympanic temperature ≥ 38 °C at least once during analgesia. Parturients with fever routinely received physical cooling and antibiotics. Demographic data such as maternal age, height, weight, gestational age, baseline cervical dilatation, white blood cell count, and neutrophils were recorded. Obstetric characteristics such as the duration of analgesia, duration of analgesia to full dilatation and delivery, duration from rupture of the membranes (ROM) to delivery, cesarean section rate, and oxytocin augmentation rate were collected. Neonatal outcomes such as neonatal weight, antibiotic usage, Apgar scores at 1 min and 5 min were also recorded. The primary outcome was the incidence of intrapartum fever, and the secondary outcomes were the duration of analgesia, analgesia to full cervical dilation and analgesia to delivery.

The sample size was estimated based on the incidence of intrapartum fever in prior studies. The risk of intrapartum fever in epidural analgesia has been reported to range from 15 to 42% [6, 17]. There were only two studies reported the risk of intrapartum fever in CSE analgesia among 7 to 14% [18, 19]. We presumed that the incidence of intrapartum fever in the epidural analgesia group was 22% and that in the CSE analgesia group was 10%. To maintain a power analysis with α = 0.05 and β = 0.2, and considering a 20% dropout rate, we calculated that the sample size to be 179 parturients per group. Finally, 200 parturient women were recruited in each group.

Statistical analysis was performed with SPSS 20.0, and *P <* 0.05 was considered significant. Continuous variables that were normally distributed are presented as the mean ± standard deviation (SD) and were analysed by Student’s t-test, while nonparametric data were analysed by Mann-Whitney U tests. Categorical variables are presented as rates and were compared by the chi-square or Fisher’s exact test. Repeated variables were analyzed by repeated measures within groups and ANOVA. Maternal temperatures were considered repeated variables and analysed with repeated measures analysis of variance.

## Results

**Fig. 1:**
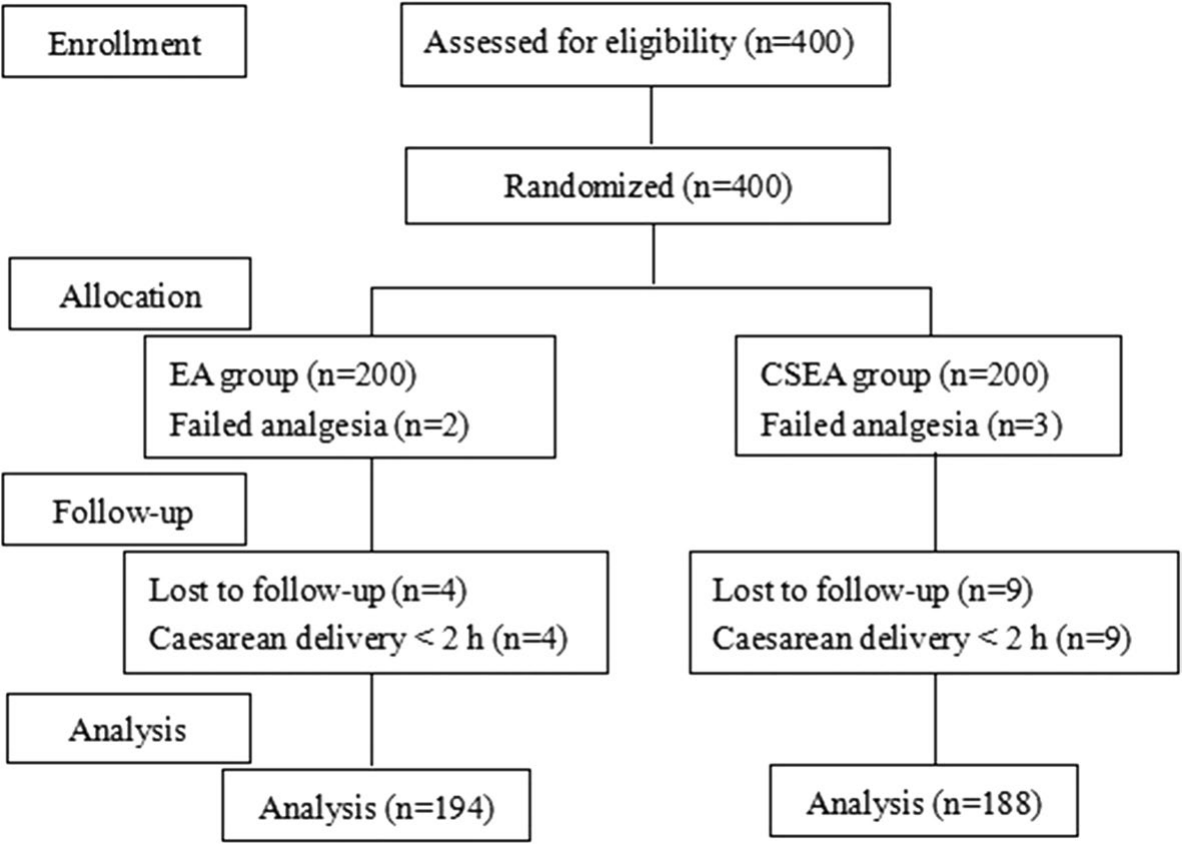
Flow chart of parturient randomized to EA and CSEA group

Four-hundred parturients participated in this study, however, 18 pregnant women were excluded, including 13 patients who had a caesarean delivery within 2 h after the initiation of analgesia and 5 patients who had failed analgesia. Finally, 382 parturients fulfilled the trial, 194 patients in the EA group and 188 patients in the CSEA group (Fig. 1). Maternal demographic characteristics such as age, weight, gestational age, and baseline maternal temperature showed no significant differences between groups. There were significant differences in height and white blood cell count between groups, but there was no clinical significance (Table 1).

**Table 1:** Maternal demographic characteristics

	EA group (*n =* 194)	CSEA group (*n =* 188)	*P*
Age (years)	29.2 ± 2.8	29.3 ± 3.2	0.751
Height (cm)	160.1 ± 5.0	161.6 ± 5.1	0.003*
Weight (kg)	65.9 ± 7.4	66.9 ± 7.8	0.203
Gestational age (weeks)	39.3 ± 0.9	39.5 ± 1.0	0.146
White blood cell count (×109 /L)	9.1 ± 2.4	8.5 ± 2.2	0.011*
Baseline maternal temperature (°C)	36.9 ± 0.2	36.9 ± 0.2	0.122

*P < 0.05 for comparison between group EA and group CSEA

**Fig. 2:**
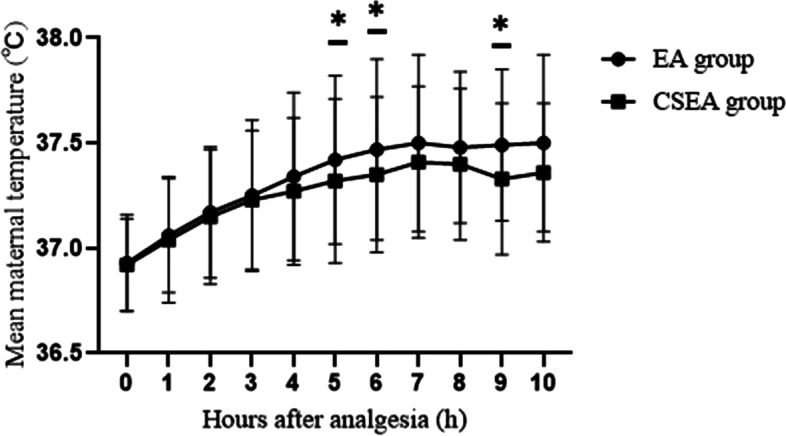
Comparisons of the mean maternal temperature after analgesia between EA group and CSEA group. All values represent the means±SD. *P < 0.05, EA Group vs CSEA Group at the time points of 5 h, 6 h, and 9 h after analgesia

**Fig. 3:**
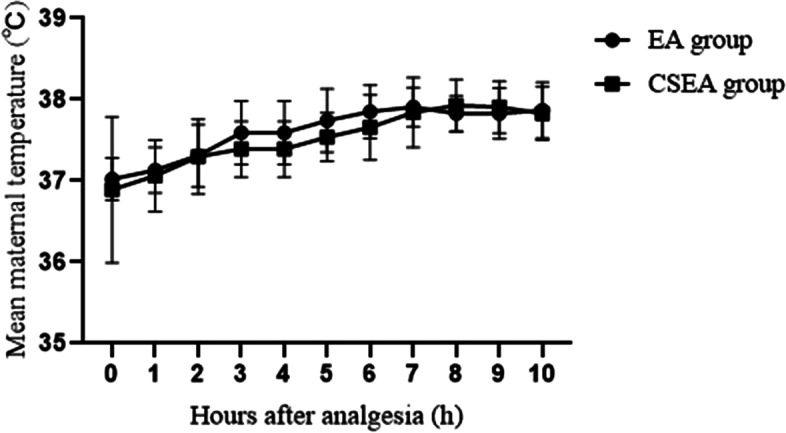
Comparisons of the mean maternal temperature after analgesia between EA group and CSEA group in the subset of women who develop fever

**Fig. 4:**
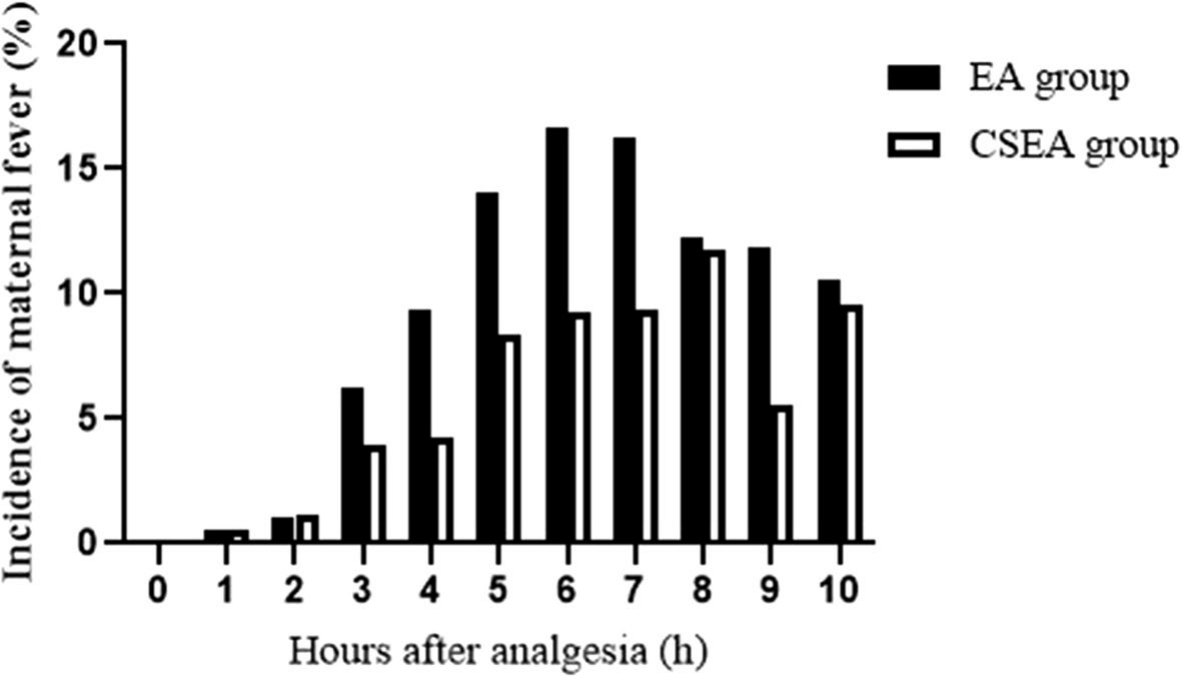
The incidence of maternal intrapartum fever (T≧38 °C) after analgesia between EA group and CSEA group

When comparing maternal temperature, the CSEA group had a lower incidence of maternal fever (14.4% VS 27.8%) than the EA group (Table 2). There was a tendency for the maternal temperature to be elevated over time during labor in both groups (*P* = 0.034). The maternal temperatures at 5 h (37.4 ± 0.3 °C VS 37.3 ± 0.03 °C), 6 h (37.5 ± 0.4 °C VS 37.4 ± 0.03 °C), and 9 h (37.5 ± 0.4 °C VS 37.3 ± 0.05 °C) after analgesia were significantly higher in the EA group than in the CSEA group (Fig. 2). We also compared the mean maternal temperature after analgesia between EA group and CSEA group in the subset of women who develop fever (Fig. 3). There was no difference between the two groups (*P* = 0.221). The incidence of maternal intrapartum fever after analgesia every hour between EA group and CSEA group was shown in Fig. 4. There was no difference between the two groups, but EA group had a tendency of high incidence.

**Table 2:** Labor events and neonatal outcomes in the two study groups

	EA group (*n =* 194)	CSEA group (*n =* 188)	*P*
Baseline cervical dilatation (cm) (median /25–75 th percentile)	2 (2–3)	2 (2–2)	0.642
Spontaneous ROM (n %)	50 (25.8%)	61 (32.4%)	0.151
Length of membrane rupture (min)	607 ± 683	627 ± 611	0.593
Oxytocin augmentation (n %)	49 (44.5%)	61 (55.5%)	0.121
Duration of analgesia (min)	504 ± 218	453 ± 210	0.022*
-Maternal fever patients	537 ± 221	542 ± 195	0.933
-No fever patients	490 ± 216	438 ± 208	0.034*
Analgesia to full cervical dilation (min)	381 ± 225	338 ± 203	0.030*
-Maternal fever patients	431 ± 225	356 ± 204	0.694
-No fever patients	375 ± 224	323 ± 198	0.05
Analgesia to delivery (min)	453 ± 240	388 ± 209	0.010*
-Maternal fever patients	513 ± 260	510 ± 182	0.955
-No fever patients	431 ± 229	373 ± 208	0.033*
Total dose of epidural local anesthetic (ml)	69 ± 30	48 ± 29	< 0.001*
-Maternal fever patients	74 ± 31	60 ± 24	0.042*
-No fever patients	66 ± 29	45 ± 29	< 0.001*
Cesarean delivery (n %)	32 (16.5%)	30 (16.0%)	0.887
Maternal fever (n %)	54 (27.8%)	27 (14.4%)	0.001*
≧38 °C (n %)	41 (75.9%)	22 (81.5%)	
≧38.5 °C (n %)	12 (22.2%)	5 (18.5%)	
≧39 °C (n %)	1 (1.9%)	0 (0%)	
Newborn weight (g)	3248 ± 335	3259 ± 358	0.755
Neonatal antibiotic use (n %)	61 (31.4)	77 (41.0)	0.053
Apgar score (1 min) (median/25-75th percentile)	10 (10–10)	10 (10–10)	0.444
Apgar score (5 min) (median/25-75th percentile)	10 (10–10)	10 (10–10)	

*P < 0.05 for comparison between group EA and group CSEA. Spontaneous ROM: Spontaneous rupture of the membranes. Length of membrane rupture: duration from rupture of the membranes to delivery

**Fig. 5:**
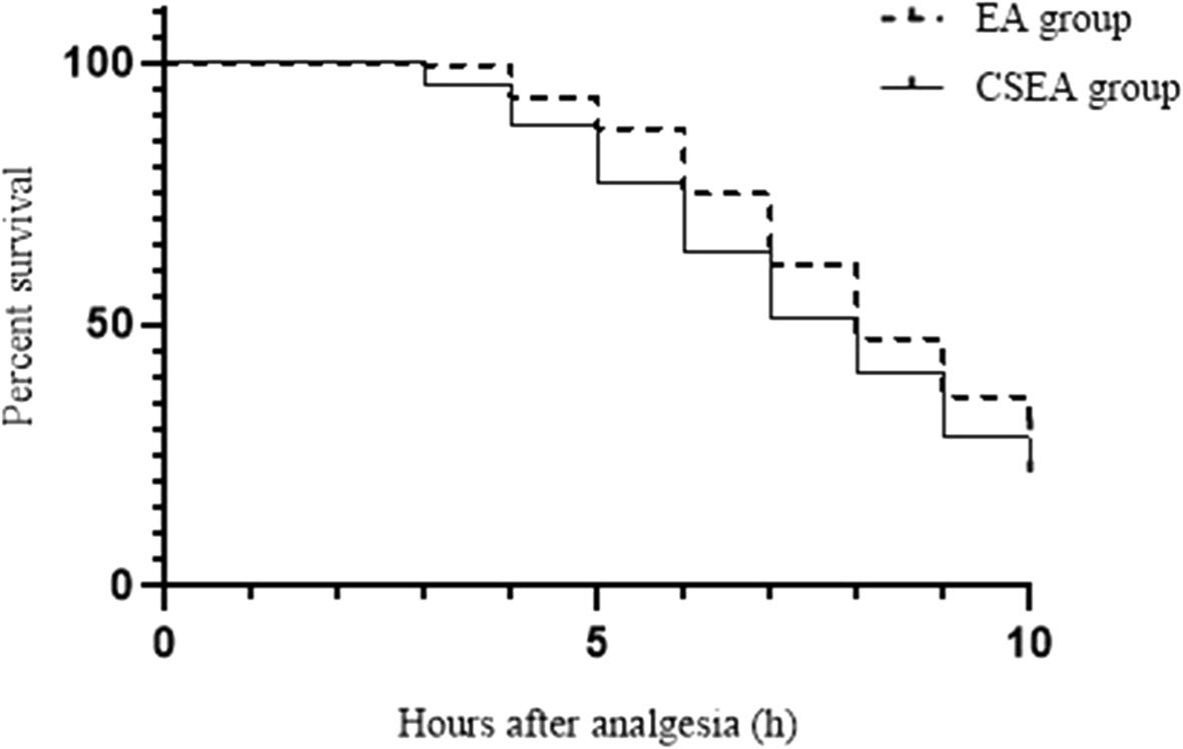
Kaplan-Meier survival curve according to maternal temperature in the EA group and CSEA group

The maternal temperature data were only analysed up to 10 h after analgesia because few parturients who had not delivered after that time. At 10 h after analgesia, there were 58 people left in the EA group and 42 people left in the CSEA group. In addition, there was no difference between the two groups about maternal temperature over 10 h after analgesia. Kaplan-Meier survival curve according to maternal temperature in the EA group and CSEA group was shown in Fig. 5.

With regard to labor events, no differences were found in the baseline cervical dilatation, length of membrane rupture, risk of spontaneous ROM or risk of cesarean delivery between the two groups. The CSEA group had a shorter duration of analgesia (453 ± 210 min VS 504 ± 218 min), analgesia to full cervical dilation (338 ± 203 min VS 381 ± 225 min) and analgesia to delivery (388 ± 209 min VS 453 ± 240 min) than the EA group. There was no difference between the CSEA group and EA group about the duration of labor analgesia, analgesia to full cervical dilation, and analgesia to delivery in the maternal fever patients. CSEA group had a shorter duration of labor analgesia and analgesia to delivery compared to the EA group in patients who did not become maternal fever. The CSEA group decreased the total dose of epidural local anesthetic (68 ± 230 ml VS 48 ± 29 ml) compared with the EA group. In relation to neonatal outcomes, no differences were observed in newborn weight, neonatal antibiotic use, 1 min Apgar score or 5 min Apgar score (Table 2).

## Discussion

The current research shows that CSEA is associated to a lower incidence of intrapartum fever, shorter duration of analgesia, and less dose of epidural local anesthetic compared to EA.

In our study, the mean maternal temperature in both groups tended to increase over time during labor. There were significant differences at the time points of 5 h, 6 h, and 9 h between the two groups. This upward trend in maternal temperature has been reported in many studies [4, 20–22]. Moreover, in this study, women who received EA had a higher risk of developing maternal fever than those who received CSEA. The incidence of maternal fever in the CSEA group was 14.4%, and for the EA analgesia group was 27.8%. The incidence rate of epidural-related fever which reported in randomized and observational studies was ranged from 15 to 42%, and the incidence in randomized trials was approximately between 22 and 33%.33 This was highly consistent with our report. There was also a study on CSEA Vs non-pharmacological methods in maternal intrapartum temperature and intrapartum fever, it was found that patients receiving CSEA experienced a significant increase in intrapartum temperature and 14% of patients developed fever [18].

The mechanism of epidural analgesia-related fever is still unclear. It has been suggested that epidural analgesia might alter maternal thermoregulation [23–25]. Parturient women can dissipate the increased heat energy through hyperventilation and perspiration in labor to maintain temperature balance. Epidural analgesia might inhibit hyperventilation due to adequate analgesia, diminished lower body sweating and reactive upper body vasoconstriction by producing a sympathetic block. Thus, disturbance of the heat production-dissipation mechanism during epidural analgesia may allow for heat retention, which causes the parturients to develop maternal fever. It is believed that increased maternal temperature during labor is not significantly associated with inflammation. In contrast, some previous studies have shown that epidural analgesia-related fever could be attributed to noninfectious inflammation, which is observed by elevated levels of maternal serum IL-6 [2, 6, 26, 27].

As reported in previous studies, epidural-related fever in labor is associated with prolonged labor. Our study showed that CSEA was associated with a shorter time of duration of analgesia, analgesia to full cervical dilation and analgesia to delivery than epidural analgesia, this was consistent with previous study outcomes. A previous study also found that CSEA resulted in a significantly more rapid cervical dilation and shorter time of full cervical dilation than EA in nulliparous parturients [16]. It was reported that CSEA decreased the duration of the first stage of labor compared with non-pharmacological methods [18]. Our study also found that there were no difference between the two groups about the duration of analgesia, analgesia to full cervical dilation and analgesia to delivery time in the maternal fever patients. There were some risk factors that might influence the fever rate such as prolonged membrane rupture, high BMI, high gestational age, and nulliparity. However, we did not see significant difference in our study. The prolonged labor time associated with EA might be attributed to the high incidence of maternal fever.

Our study also found that the total dose of epidural local anesthetic was decreased in the CSEA group than in the EA group. For the sub-analyze of maternal fever patients, CSEA group also had less dose of epidural local anesthetic than EA group. Recent studies suggested that the noninfectious maternal fever may be related to the effects of ropivacaine/bupivacaine on inflammatory mediators or inhibitors [28, 29]. The more local anesthetic used in the EA group might be attributed to a possible mechanism of maternal fever. Our study found that maternal fever was not indicative of any increased risk of adverse neonatal outcomes, which is consistent with other studies [30].

There are some limitations to our study. It is not a double-blind trial since the two groups using a distinctly different method. However, we think that the unblended nature of the study will not affect the temperature data which is objective and is the primary outcome of our study. Moreover, the side effects of analgesia such as fetal heart rate (FHR) abnormalities and pruritus were not recorded. Besides, we assumed that all maternal fever is epidural-related maternal fever. However, this situation was probably not, especially for longer labors when the development of chorioamnionitis was more likely.

## Conclusion

In conclusion, our data show that maternal temperature gradually increased over time with both types of analgesia during labor. CSEA was associated with a lower incidence of intrapartum fever, shorter duration of analgesia, and less dose of epidural local anesthetic compared to EA in nulliparous women.

## Data Availability

The datasets are not publicly available, but available from the corresponding author on reasonable request.

## Abbreviations

EA: Epidural analgesia
CSEA: Combined spinal-epidural analgesia
ROM: Duration from rupture of the membranes
